# Self-learning GAN based synthetic CT generation: unlocking CBCT-based adaptive radiotherapy

**DOI:** 10.3389/fonc.2026.1756153

**Published:** 2026-02-20

**Authors:** Jessica Prunaretty, Lorenzo Colombo, Sami Romdhani, Olivier Teboul, David Azria, Nikolaos Paragyios, Pascal Fenoglietto

**Affiliations:** 1 Department of Radiation Oncology, Institut du Cancer de Montpellier, Montpellier, France; 2 Clinical affairs, TheraPanacea, Paris, France; 3 AI Engineering, TheraPanacea, Paris, France; 4 Chief Executive Officer (CEO), TheraPanacea, Paris, France

**Keywords:** adaptive radiotherapy, artificial intelligence, CBCT, deep learning, synthetic CT

## Abstract

**Purpose/objectives:**

This study proposes and clinically evaluates synthetic CT (sCT) images generated from multi-center CBCT scans using artificial intelligence, with the aim of fully leveraging CBCT for adaptive radiotherapy in patients with pelvic, head-and-neck, lung, and breast cancer.

**Materials and methods:**

In collaboration with TheraPanacea (Paris, France), AI-based sCT models were developed for multiple anatomical sites using a cycleGAN architecture. The study included 51 patients from two European institutions diagnosed with head-and-neck, lung, pelvic or breast cancer and treated with CBCT-based position verification. CBCT scans were acquired using two linear accelerator systems (Varian and Elekta). Image accuracy was assessed using MAE, SSIM, and PSNR. For dosimetric evaluation, planning CTs (pCTs) were non-rigidly registered to CBCTs. Treatment plans were created on the pCT using a clinical TPS to meet standard clinical criteria, then recalculated on both the warped CT (wCT) and sCT. Dose distributions were compared using global gamma passing rates and dose-volume metrics.

**Results:**

The proposed model substantially improved image quality compared with CBCT. MAE decreased from 122.95 ± 50.07 to 23.65 ± 10.09, while SSIM increased from 0.78 ± 0.12 to 0.97 ± 0.03 and PSNR from 35.01 ± 7.24 to 44.35 ± 7.07. Dose-metric comparisons showed strong agreement between the pCT and wCT, with median relative differences within 0.5% for both targets and organs at risk. Median gamma passing rates for 2%/2 mm and 3%/3 mm criteria (10% threshold) reached 100% across all anatomical sites. No performance differences were observed between Elekta- and Varian-sCTs.

**Conclusion:**

This multi-center study demonstrates the feasibility of generating clinically acceptable AI-based sCTs from CBCT for multiple anatomical sites, yielding consistent image quality improvements and reliable dosimetric accuracy.

## 1 Introduction

Online adaptive radiotherapy (oART) is becoming more widely used in clinical practice worldwide due to its ability to re-optimize treatment plans to account for daily patient-specific treatment variations (1, 2). The first clinical workflow programs for online ART were established using magnetic resonance guided radiotherapy (MRgRT) (3, 4) and Ethos^®^ Therapy was the first oART solution commercialized by Varian Medical System using cone beam computed tomography (CBCT) images (5).

The use of CBCT for dose calculation holds significant appeal when it comes to dose reporting and monitoring, particularly in the context of dose-guided adaptive radiotherapy. Although CBCT has revolutionized image-guided radiotherapy (IGRT) and is extensively used for daily patient setup (6), its use for plan adaptation is still limited. The main reasons are the artefacts related with scatter and the reconstruction algorithms, which reduce image quality and CBCT-based dose calculation accuracy (7–9). Several solutions have been proposed using deformable image registration (10–12), scatter correction (13, 14), or rescaling of Hounsfield unit intensities (15).

With the development of artificial intelligence, studies have focused on deep learning approaches (16–19). Kia et al. (20) was the first team to create a so-called synthetic-CT from a CBCT. They developed a deep convolutional neural network method for improving CBCT image quality for prostate cancer patients. Since then, the number of investigations have grown, resulting in many different architectures. In the recent years, the generative adversarial network (GAN) is one of the most developed architectures due to the anatomical preservation and the use of unpaired data sets. Xue et al. (21) generated sCT from head and neck CBCT using the CycleGAN, Pix2Pix and U-Net models and the performance of CycleGAN was proved to be best among three models in terms of image quality and dose accuracy. Likewise, Pang et al. (22) compared four deep learning architectures (cycleGAN, Unet, Unet + cycleGAN, conditional GAN) for generating nasopharynx CBCT and showed the superiority of the conditional GAN model in an adaptive proton treatment planning application. Maspero et al. (23) also demonstrated the feasibility to use a single cycle-GAN architecture for different anatomical sites. However, few studies investigated the model training using multi-center datasets (24) as proposed for MR-to-CT synthesis (25, 26). This study introduces and evaluates an artificial intelligence–based method for synthetic CT (sCT) generation in a pre-clinical validation setting to address these challenges, with the aim of fully harnessing the potential of CBCT for adaptive radiotherapy in patients with pelvic, head and neck, lung, and breast cancers.

## 2 Materials and methods

### 2.1 Deep learning workflow

Cycle generative adversarial networks (CycleGANs) are deep learning architectures designed to learn bijective transformations between image modalities. Training a model to generate CT images from CBCTs is challenging due to the intrinsic difficulty of acquiring perfectly aligned CBCT–CT pairs. To overcome this limitation, a two-stage cycle architecture combined with a patented CBCT simulation approach. was developed.

In the first stage, perfectly aligned CBCT–CT pairs were generated by simulating CBCT acquisitions directly from real CT volumes. This simulation reproduces the physical principles of clinical CBCT imaging, in which image quality degradation primarily arises from the limited number of projections compared to conventional CT. Specifically, simulated CBCTs were obtained by projecting a reduced number of cone-beam projections from CT volumes onto integral planes, followed by 3D reconstruction using an inverse Radon transform based on the method described by Biguri et al. (27). By construction, these simulated CBCTs are fully aligned with their corresponding CTs, thereby eliminating registration inaccuracies and enabling robust supervised training.

In the second stage, the model was refined using real CBCT data, allowing the network to adapt to clinical image characteristics while preserving spatial consistency. The GAN was thus trained to generate synthetic CTs from real CBCTs that became indistinguishable from true CT images. This two-stage strategy, combining physics-based CBCT simulation with cycle-consistent adversarial learning, distinguishes our approach from previously reported models that rely on imperfectly registered CBCT–CT datasets or purely unsupervised training.

The training and validation dataset comprised 1063 planning CTs and 228 CBCTs for head and neck, 278 planning CTs and 174 CBCTs for breast, 484 planning CTs and 324 CBCTs for thorax, and 317 planning CTs with 194 CBCTs for pelvis. Data were provided by three European institutions (referred to as Centers A, B and C) and were split into training (80%) and validation (20%) sets. Details of simulated CBCTs derived from planning CTs, stratified by anatomy, center, and CBCT vendor, as well as imaging protocols, are reported in **Supplementary Material 1**.

### 2.2 Augmentation of the synthetic CT

As the CBCT field of view (FOV) is smaller than that of the planning CT, the synthetic CT (sCT) is extended using information from the planning CT to enable organ-at-risk segmentation and dose computation. The augmentation is performed in the X, Y, and Z directions, with particular emphasis on the superior–inferior axis. First, the CBCT FOV and the external contours of both the sCT and the planning CT are detected. A deformable registration is then computed between the planning CT and the sCT. From this registration, a variable-weight mask is generated, assigning a weight of 1 inside the sCT external contour and an exponentially decreasing weight outside the contour. The deformable displacement field is resampled using this mask, resulting in a hybrid transformation: near the sCT FOV, the deformation corresponds to the deformable registration, ensuring geometric continuity, while far from the sCT FOV the deformation progressively vanishes, effectively reverting to a rigid transformation and thus minimizing assumptions on patient geometry.

The planning CT and its contours are warped using this resampled displacement field, followed by a rigid registration between the sCT and the warped planning CT (wCT). Finally, the superior and inferior boundary slices of the sCT are identified, and slices from the wCT are appended above and below these boundaries to obtain an augmented synthetic CT with a full planning CT FOV and without geometric discontinuities.

### 2.3 Patient selection

An independent, retrospective cohort of 10 prostate, 16 head and neck, 12 lung and 13 breast cancer patients treated at two leading European cancer treatment centers (referred to as Centers B and D) was selected for this evaluation. These testing datasets were entirely different from the cycle-GAN model training datasets. CBCT scans were performed using equipment from two different linac manufacturers, Varian and Elekta. For each patient, a single CBCT fraction was randomly selected and used for sCT generation and comparison with the planning CT.

### 2.4 Image accuracy

Mean absolute error (MAE), structural similarity index measure (SSIM), peak signal to noise ratio (PSNR) were computed to assess the image accuracy. The MAE quantifies the average absolute pixel-wise difference between the sCT and the reference CT, defined as:


$$MAE=\frac{1}{N}\sum_{i=1}^{N}\left|x_{i}-y_{i}\right|$$

where x_i_ and y_i_ represent pixel intensities of the ground truth and synthetic images, respectively, and N is the total number of pixels. Lower MAE values indicate higher accuracy.

The SSIM assesses perceptual similarity by comparing luminance, contrast, and structural information between the two images, and is given by:


$$SSIM=\;\frac{\left(2\mu_{x}\mu_{y}+C_{1}\right)\left(2\sigma_{xy}+C_{2}\right)}{\left(\mu_{x}^{2}+\mu_{y}^{2}+C_{1}\right)\left(\sigma_{x}^{2}+\sigma_{y}^{2}+C_{2}\right)}$$

where μ_x_, μ_y_ are mean intensities, σ_x_, σ_y_ are standard deviations, σ_xy_ is the covariance, and C_1_, C_2_ are small constants to prevent division instability SSIM values range from 0 (no similarity) to 1 (perfect similarity).

The PSNR measures image fidelity relative to the maximum possible intensity value, expressed as:


$$PSNR=20\log_{10}\left(\frac{MAX_{pix}}{\sqrt{MSE}}\right)$$

where MAX_pix_ is the maximum pixel intensity and MSE is the mean squared error. Overall, lower MAE and higher SSIM and PSNR values indicate closer correspondence and improved agreement between the sCT and the reference CT.

To evaluate the Hounsfield Unit (HU) differences between sCT and CT images, the planning CT images were deformably registered to the corresponding CBCT images to generate reference CT datasets for testing. The synthetic CT images were subsequently compared with the CBCT images to assess the reconstruction performance. All similarity metrics were computed within the CBCT-defined mask.

### 2.5 Dosimetric evaluation

Planning CTs were deformably registered to the CBCTs for each patient to account for changes in body anatomy and positioning. Treatment plans were optimized on the wCT and recalculated on the sCTs for the dosimetric evaluation. Dose calculations were performed using the AAA algorithm within Eclipse TPS (version 15.6, Varian Medical Systems, Palo Alto, CA, USA) in a volumetric modulated arc therapy (VMAT) configuration using a dose calculation grid size of 0.25 cm. Dose prescriptions were detailed in **Supplementary Material 1**.

For analysis, wCT and sCT were compared using DVH parameters for PTV and organs at risk. Given the variations in dose prescriptions, the dose comparison was conducted using the relative dose difference, which is defined as:


$$\Delta D_{x}=\frac{D_{x}^{sCT}-D_{x}^{wCT}}{D_{x}^{wCT}}$$

With $D_{x}^{sCT}$ the dose metric from the sCT and $D_{x}^{wCT}$ the dose metric from the wCT. Reference structures for DVH parameters were rigidly transferred from the planning CT to sCT. In the case of simultaneous integrated boost treatment, the larger PTV was retained. Dose metrics are detailed in **Table 1**. In addition, 3D dose distributions were evaluated using global gamma criteria (2%/2mm and 3%/3mm) with a 10% threshold using VeriSoft software (PTW Dosimetry, Freiburg, Germany). The 3%/3 mm and 2%/2 mm criteria were selected to enable comparison with the existing literature.

**Table 1:** Dose metrics used for the dosimetric evaluation.

Prostate	PTV	D_2%_	Thorax	PTV	D_2%_
		D_50%_			D_50%_
		D_95%_			D_95%_
		D_98%_			D_98%_
	Rectum	D_50%_		Spinal cord	D_0.05cc_
		D_25%_		Lung	V_20Gy_
		D_mean_			D_mean_
	Bladder	D_50%_		Heart	V_30Gy_
		D_25%_			D_mean_
		D_mean_	Breast	PTV	D_2%_
Head & Neck	PTV	D_2%_			D_50%_
		D_50%_			D_95%_
		D_95%_			D_98%_
		D_98%_		Spinal cord	D_0.05cc_
	Spinal cord	D_0.05cc_		Lung	V_20Gy_
	Parotid	D_mean_			D_mean_
	Brainstem	D_1cc_		Heart	V_30Gy_
	Larynx	D_mean_			

## 3 Results

Training of the synthetic CT model required approximately two weeks. The images were resampled to an isotropic resolution of 1 mm³ and cropped or padded to a fixed size of 16 × 512 × 512 voxels (Z, Y, X). Model convergence was achieved after approximately 200 training epochs using NVIDIA RTX 3090 GPUs. Using two GPUs, the generation of a sCT image took about 30 seconds, with an additional 20 seconds for augmentation, resulting in a total generation time of roughly 50 seconds per case. Generation time may vary depending on hardware configuration and imaging parameters.

**Figure 1:**
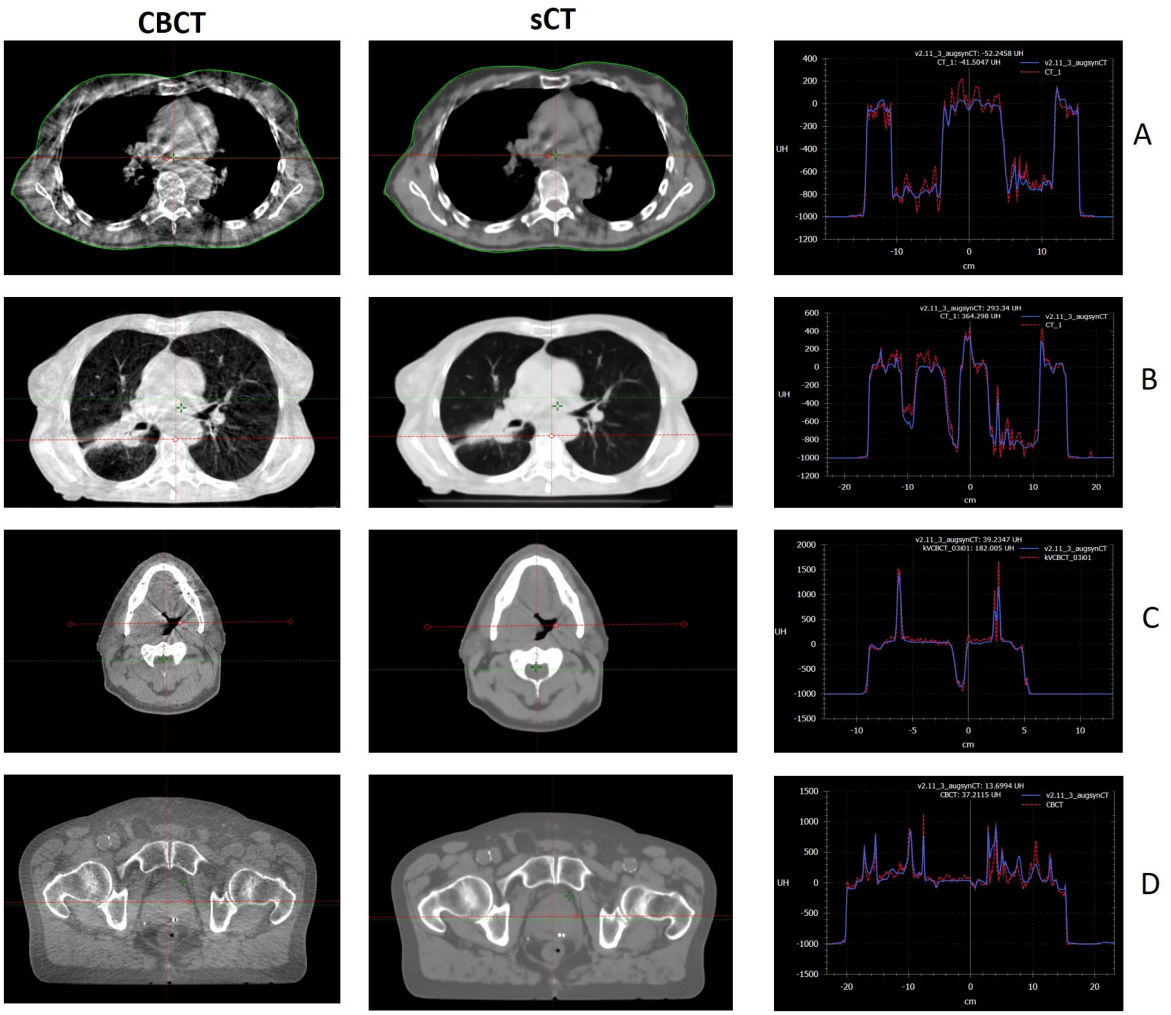
Example of sCT (right) and the CBCT (left) images for breast **(A)**, thorax **(B)**, head and neck **(C)** and male pelvis **(D)** anatomies. Example of Hounsfield unit comparison between sCT (blue line) and CBCT (red line).

**Figure 1** presents representative examples of sCTs generated by the AI-based model, illustrating the comparison of Hounsfield Units with the corresponding deformably registered CT images for the male pelvis, thorax, head and neck, and breast regions. The image quality of sCTs was markedly improved compared with CBCT, primarily due to reduced scatter artifacts and smoother the HU distributions.

The **Table 2** summarizes the quantitative image similarity metrics for each anatomical site. Overall, the MAE decreased from 122.95 ± 50.07 to 23.65 ± 10.09, the SSIM increased from 0.78 ± 0.12 to 0.97 ± 0.03 and the PSNR increased from 35.01 ± 7.24 to 44.35 ± 7.07 when comparing CBCT and sCT images. Despite variations in CBCT image quality between Varian and Elekta acquisitions, only minor differences were observed in the corresponding sCT reconstructions (**Table 3**).

**Table 2:** Comparison of MAE, SSIM and PSNR between CBCT and sCT images for breast, thorax, head and neck and male pelvis localizations.

Anatomical site	Image type	MAE (HU)	SSIM	PSNR
Breast	CBCT	95.54 ± 23.30	0.79 ± 0.08	35.53 ± 5.51
	sCT	25.99 ± 7.51	0.97 ± 0.03	43.54 ± 7.10
Thorax	CBCT	104.12 ± 31.03	0.73 ± 0.22	33.10 ± 9.64
	sCT	32.74 ± 9.43	0.97 ± 0.02	43.12 ± 4.34
Head&Neck	CBCT	169.38 ± 58.13	0.81 ± 0.14	36.51 ± 6.95
	sCT	15.11 ± 6.52	0.98 ± 0.02	47.75 ± 7.33
Male pelvis	CBCT	98.79 ± 36.24	0.74 ± 0.16	34.88 ± 6.21
	sCT	23.39 ± 6.15	0.97 ± 0.03	41.44 ± 6.82
Global	CBCT	122.95 ± 50.07	0.78 ± 0.12	35.01 ± 7.24
	sCT	23.65 ± 10.09	0.97 ± 0.03	44.35 ± 7.07

**Table 3:** Comparison of MAE, SSIM, and PSNR for CBCT and sCT images across different anatomical regions (breast, thorax, head and neck, and male pelvis).

		Breast			Thorax			Head & neck			Male pelvis		
Image type	Manufacturer	MAE	SSIM	PSNR	MAE	SSIM	PSNR	MAE	SSIM	PSNR	MAE	SSIM	PSNR
CBCT	Varian	78.60	0.81	36.05	96.32	0.72	30.97	103.93	0.87	40.94	67.01	0.81	36.66
	Elekta	115.30	0.78	34.92	121.24	0.83	39.65	208.65	0.77	33.85	130.56	0.67	33.10
sCT	Varian	20.96	0.98	46.03	32.51	0.96	39.65	13.47	0.99	50.48	22.03	0.97	41.56
	Elekta	31.86	0.95	40.63	32.90	0.98	45.60	16.09	0.98	46.11	24.76	0.96	41.32

Results are reported separately for images acquired with Varian and Elekta systems.

**Figure 2:**
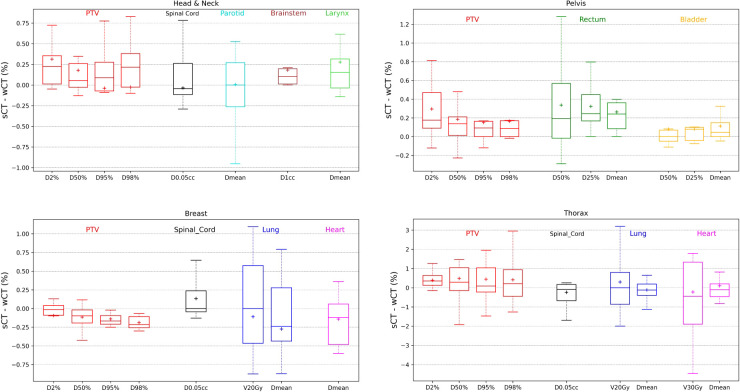
Relative dose deviations between the synthetic CT and the warped CT for the PTV and organs at risk using different dose metrics for male pelvis, thorax, head and neck and breast localizations.

**Figure 3:**
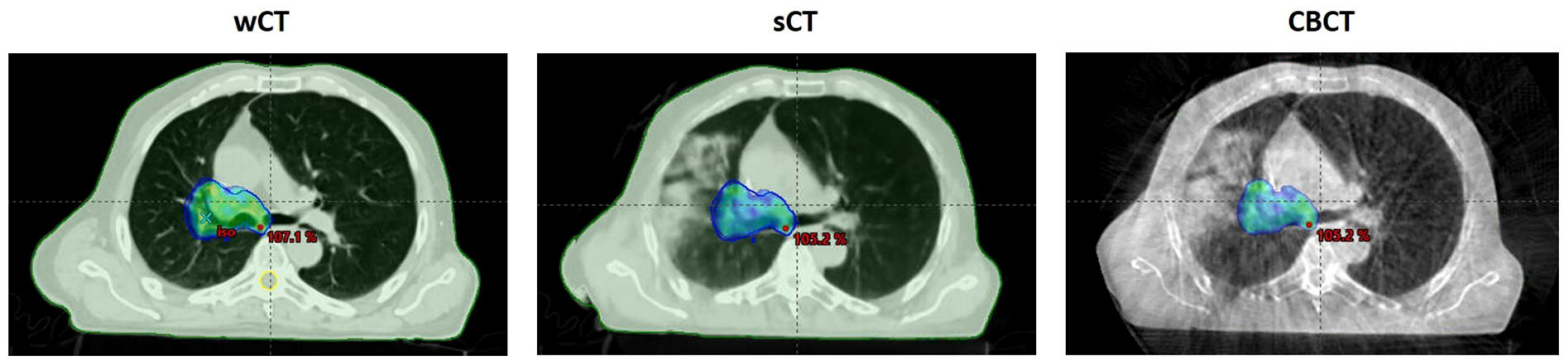
Example of comparison of dose distribution (95% isodose) calculated on the wCT (left), sCT (center) and displayed on the CBCT (right) for a patient treated for a lung cancer.

**Figure 2** shows the relative dose difference for the PTV and the organs at risk between plans calculated using the synthetic CT and the warped CT for pelvis, thorax, head and neck and breast localizations. The median relative dose differences for the PTV were lower than 0.5% for each dose metric (D_98%_, D_95%_, D_50%_, and D_2%_). The maximum reported value was 2.95% for D_98%_, equivalent to 1.05Gy for the thorax. Concerning the organs at risk, the median relative dose difference were less than 0.5%, regardless the dose metrics and localizations assessed. The maximal difference was -4.46% for V30Gy of the heart, corresponding to 0.10Gy. The largest relative dose difference for PTV and heart are from the same patient illustrated in **Figure 3**.

The median global gamma analysis using two tolerance criteria (3%/3 mm and 2%/2 mm) with a 10% dose threshold demonstrated strong agreement between the dose distributions calculated on the synthetic CT and the warped CT (**Table 4**). The lowest gamma passing rate was observed for the thorax case, with a value of 96.6% for the 2%/2 mm criterion. This corresponded to the same patient presented earlier (**Figure 3**).

**Table 4:** Comparison of the global gamma passing rates for pelvis, thorax, head and neck and breast localizations using 3%3mm and 2%2mm criterion with 10% dose thresholds.

	Gamma pass rates (Median [Min-Max])	
Anatomical site	3%3mm	2%2mm
Pelvis	100 [99.6 - 100]	100 [99.2 - 100]
Thorax	100 [99.7 - 100]	100 [96.6 - 100]
Head & Neck	100 [98.7 - 100]	100 [97.4 - 100]
Breast	100 [100]	100 [99.9 - 100]

## 4 Discussion

This study introduced 3D cycle GAN models for generating synthetic-CTs from CBCT images in cases of pelvic, head and neck, lung, and breast cancer. The key strengths of this work include its multi-centric approach, incorporating CBCT images from various linac manufacturers with differing characteristics, and the extensive patient dataset used for model training, ensuring robust synthetic-CT generation. Additionally, the cycle GAN architecture’s ability to function without paired images is particularly beneficial for areas like the pelvis, where anatomical variations are frequent.

Regarding the qualitative evaluation, the results obtained in this study are satisfying, showing a good agreement between the synthetic-CT and the warped CT. In addition, this research demonstrated that AI-driven synthetic CT generation from CBCT is clinically accurate across multiple anatomical sites. In terms of image accuracy, the MAE was 23, 65 ± 10, 09 for all confounded localizations. Dosimetric assessment showed median dose differences within 0.5% and median gamma pass rates of 100% for both the 3%/3mm and 2%/2mm criteria. Our results are consistent with recent literature that extends image quality analysis to include additional dose assessment (28–32). For example, O’Hara et al. (30) evaluated the image and dose accuracy of AI-guided sCTs generated from head and neck CBCT images. They reported a MAE of 79.4 HU, a gamma pass rate of 99.8% (2%/2mm, 20% dose threshold) and a generation time of 112 seconds. However, their dosimetric evaluation was limited to the CBCT field of view, which does not encompass the entire patient volume, potentially limiting its effectiveness in an adaptive workflow. Xie et al. (31) developed a technique similar to ours for handling truncated CBCTs in breast cancer cases and demonstrated excellent dose calculation accuracy with gamma pass rates of 98.98 ± 0.64% and 99.69 ± 0.22% for 2%/2mm and 3%/3mm criteria, respectively.

A primary limitation of this study is that the planning CT was used as the ‘ground truth’ to evaluate the quality of the sCTs generated from CBCT data. Despite using a wrapped CT to reduce discrepancies, anatomical variations may still be introduced by the time interval between the planning CT and the CBCT acquisition, such as differences in organ filling, air cavity fluctuations, or tumour regression, that can affect the agreement between the two image sets. Consequently, as illustrated in **Figure 3**, some of the observed deviations may be due to imperfections in the reference images rather than to limitations in the sCT generation process itself. Furthermore, although differences in CBCT image quality were observed between the Varian and Elekta systems, these did not appear to cause any noticeable deterioration in the corresponding sCTs. However, the relatively small number of test cases per anatomical site and per manufacturer reduces the strength and generalisability of these findings. Therefore, expanding the patient cohort in future studies will be essential to improve the statistical robustness of the conclusions. Finally, the sCT generation framework was evaluated in a pre-clinical validation setting and was not clinically deployed at the time of the study. Consequently, the results reflect a controlled research environment and may not fully capture the constraints and variability encountered in routine clinical workflows. While deformable image registration between planning CT and CBCT is a mature and commercially available approach for adaptive radiotherapy (Ethos, Raysearch, RadFormation, SeeTreat, …), deep learning–based sCT generation remains an emerging technique with limited clinical implementation (30). Therefore, the reproducibility and generalizability of the present findings will require confirmation through prospective clinical studies, including assessment of robustness, workflow integration, and regulatory considerations, before widespread clinical adoption can be considered.

The growing number of sCT generators developed using different deep learning methods presents a challenge too, as these networks are often trained on different datasets and anatomies, and evaluated using different metrics. This inconsistency makes it difficult to perform a standardized methodological comparison, which in turn hinders the ability to identify the optimal network design choices for clinical sCT tools. In this context, the SynthRAD2023 challenge (covering brain and pelvic patients), followed by SynthRAD2025 (covering head-and-neck, thorax, and abdomen), was designed to compare and benchmark synthetic CT generation methods using multi-center ground truth data (33–35). The SynthRAD2023 findings indicated no significant correlation between image similarity metrics and dosimetric accuracy, thereby highlighting the necessity of comprehensive dose evaluation when assessing the clinical viability of sCT in radiotherapy workflows (33). Furthermore, the absence of standardized criteria for assessing and reporting sCT quality has been recognized as a key factor impeding the clinical adoption of new methods (36). Developing a robust quality assurance process will be a future challenge to ensure the safe and reliable integration of deep learning technologies into clinical workflows (37).

## 5 Conclusion

This retrospective multi-center study has shown the potential of synthetic CT across various anatomical sites. This AI-based tool is clinically acceptable, allows for significantly faster image conversion, and may enhance the implementation of adaptive radiotherapy treatments.

## Data Availability

The raw data supporting the conclusions of this article will be made available by the authors, without undue reservation.
