# Supplementary material for: Self-learning GAN based synthetic CT generation: unlocking CBCT-based adaptive radiotherapy
Source: Front Oncol. 2026 Feb 20;16:1756153. doi: 10.3389/fonc.2026.1756153 (PMC12963003; doi:10.3389/fonc.2026.1756153)
Supplement: Supplementary file 1 [file Table1.docx]

Supplementary Material

# Supplementary Data

|  | Anatomy | Number of simulated CBCTs | Slice thickness Min-Max | KVp | Rows | Columns |
| --- | --- | --- | --- | --- | --- | --- |
| Center A (Varian) | Pelvis M | 111 | 2,5 mm | 120 | 512 | 512 |
|  | Thorax M | 8 | 2,5 mm | 120 | 512 | 512 |
|  | Thorax F | 14 | 2,5 mm | 120 | 512 | 512 |
|  | Head | 26 | 2,5 mm | 120 | 512 | 512 |
|  | Neck | 82 | 1,25-2,5 mm | 120 | 512 | 512 |
| Center B (Elekta) | Pelvis M | 85 | 1,25-2,5 mm | 120 | 512 | 512 |
|  | Thorax M | 60 | 1,25-2,5 mm | 120 | 512 | 512 |
|  | Thorax F | 94 | 2,5 mm | 120 | 512 | 512 |
|  | Head | 149 | 1,25-2,5 mm | 120 | 512 | 512 |
|  | Neck | 150 | 1,25-2,5 mm | 120 | 512 | 512 |
| Center C (Varian) | Pelvis M | 121 | 1-3 mm | 120 | 512 | 512 |
|  | Thorax M | 138 | 2-3 mm | 120 | 512 | 512 |
|  | Thorax F | 170 | 1-3 mm | 120 | 512 | 512 |
|  | Head | 306 | 1-3 mm | 120 | 512 | 512 |
|  | Neck | 350 | 1-3 mm | 120 | 512 | 512 |

Table 1: Description of the data used for model training and validation. Details of simulated CBCTs derived from planning CTs are reported by anatomy and center (including the associated CBCT vendor), together with the corresponding imaging protocols. Note that the Head and Neck model was trained using Head and Neck data, the Breast model was trained using female thorax (Thorax F) data, and the Thorax model was trained using both female and male thorax (Thorax F and M) data.

|  | Patient n° | Prescription | Fraction | Manufacturer | Tension | mA | Slice thickness | Rows | Columns | Pixel spacing |
| --- | --- | --- | --- | --- | --- | --- | --- | --- | --- | --- |
| Breast | 1 | 63.22Gy SIB | 29Fr | Varian | 125 kV | 20 | 2 mm | 512 | 512 | 0.908\0.908 |
|  | 2 | 63.22Gy SIB | 29Fr | Varian | 125 kV | 20 | 2 mm | 512 | 512 | 0.908\0.908 |
|  | 3 | 63.22Gy SIB | 29Fr | Varian | 125 kV | 20 | 2 mm | 512 | 512 | 0.908\0.908 |
|  | 4 | 50Gy SIB | 25Fr | Varian | 125 kV | 15 | 2 mm | 512 | 512 | 0.908\0.908 |
|  | 5 | 63.22Gy SIB | 29Fr | Varian | 125 kV | 15 | 2 mm | 512 | 512 | 0.908\0.908 |
|  | 6 | 52.2Gy SIB | 29Fr | Varian | 125 kV | 15 | 2 mm | 512 | 512 | 0.908\0.908 |
|  | 7 | 52.2Gy SIB | 25Fr | Varian | 125 kV | 15 | 2 mm | 512 | 512 | 0.908\0.908 |
|  | 8 | 52.2Gy SIB | 29Fr | Elekta | 120 kV | 20 | 1 mm | 410 | 410 | 1\1 |
|  | 9 | 63.22Gy SIB | 29Fr | Elekta | 120 kV | 20 | 1 mm | 410 | 410 | 1\1 |
|  | 10 | 52.2Gy SIB | 29Fr | Elekta | 120 kV | 20 | 1 mm | 410 | 410 | 1\1 |
|  | 11 | 52.2Gy SIB | 29Fr | Elekta | 120 kV | 20 | 1 mm | 410 | 410 | 1\1 |
|  | 12 | 52.2Gy SIB | 29Fr | Elekta | 120 kV | 20 | 1 mm | 410 | 410 | 1\1 |
|  | 13 | 52.2Gy SIB | 29Fr | Elekta | 120 kV | 20 | 1 mm | 410 | 410 | 1\1 |
| Head & Neck | 1 | 60Gy SIB | 30Fr | Varian | 100 kV | 30 | 2 mm | 512 | 512 | 0.550\0.550 |
|  | 2 | 55Gy SIB | 20Fr | Varian | 100 kV | 30 | 2 mm | 512 | 512 | 0.550\0.550 |
|  | 3 | 55Gy SIB | 20Fr | Varian | 100 kV | 30 | 2 mm | 512 | 512 | 0.550\0.550 |
|  | 4 | 70Gy SIB | 33Fr | Varian | 100 kV | 30 | 2 mm | 512 | 512 | 0.550\0.550 |
|  | 5 | 70Gy SIB | 35Fr | Varian | 100 kV | 30 | 2 mm | 512 | 512 | 0.550\0.550 |
|  | 6 | 70Gy SIB | 35Fr | Varian | 100 kV | 15 | 2 mm | 512 | 512 | 0.511\0.511 |
|  | 7 | 50Gy SIB | 25Fr | Elekta | 120 kV | 20 | 1 mm | 410 | 410 | 1\1 |
|  | 8 | 60Gy | 30Fr | Elekta | 100 kV | 10 | 1 mm | 410 | 410 | 1\1 |
|  | 9 | 60Gy | 30Fr | Elekta | 120 kV | 20 | 1 mm | 410 | 410 | 1\1 |
|  | 10 | 70Gy SIB | 35Fr | Elekta | 100 kV | 10 | 1 mm | 410 | 410 | 1\1 |
|  | 11 | 70Gy SIB | 35Fr | Elekta | 100 kV | 10 | 1 mm | 410 | 410 | 1\1 |
|  | 12 | 60Gy SIB | 30Fr | Elekta | 100 kV | 10 | 1 mm | 410 | 410 | 1\1 |
|  | 13 | 60Gy SIB | 30Fr | Elekta | 100 kV | 10 | 1 mm | 410 | 410 | 1\1 |
|  | 14 | 60Gy | 30Fr | Elekta | 100 kV | 10 | 1 mm | 270 | 270 | 1\1 |
|  | 15 | 60Gy | 30Fr | Elekta | 100 kV | 10 | 1 mm | 270 | 270 | 1\1 |
|  | 16 | 60Gy | 30Fr | Elekta | 120 kV | 20 | 1 mm | 410 | 410 | 1\1 |
| Lung | 1 | 40Gy | 20Fr | Varian | 125 kV | 35 | 2 mm | 512 | 512 | 0.962\0.962 |
|  | 2 | 60Gy | 30Fr | Varian | 125 kV | 35 | 2 mm | 512 | 512 | 0.962\0.962 |
|  | 3 | 40Gy | 20Fr | Varian | 125 kV | 35 | 2 mm | 512 | 512 | 0.962\0.962 |
|  | 4 | 66Gy | 33Fr | Varian | 125 kV | 15 | 2 mm | 512 | 512 | 0.962\0.962 |
|  | 5 | 40Gy | 20Fr | Varian | 125 kV | 35 | 2 mm | 512 | 512 | 0.962\0.962 |
|  | 6 | 60Gy | 30Fr | Elekta | 120 kV | 20 | 1 mm | 410 | 410 | 1\1 |
|  | 7 | 60Gy | 30Fr | Elekta | 120 kV | 20 | 1 mm | 410 | 410 | 1\1 |
|  | 8 | 60Gy | 30Fr | Elekta | 120 kV | 20 | 1 mm | 410 | 410 | 1\1 |
|  | 9 | 52.2Gy SIB | 29Fr | Elekta | 120 kV | 20 | 1 mm | 410 | 410 | 1\1 |
|  | 10 | 60Gy | 30Fr | Elekta | 120 kV | 20 | 1 mm | 410 | 410 | 1\1 |
|  | 11 | 60Gy | 30Fr | Elekta | 120 kV | 20 | 1 mm | 410 | 410 | 1\1 |
|  | 12 | 60Gy | 30Fr | Elekta | 120 kV | 20 | 1 mm | 410 | 410 | 1\1 |
| Pelvis | 1 | 80Gy SIB | 40Fr | Varian | 125 kV | 33 | 2 mm | 512 | 512 | 0.908\0.908 |
|  | 2 | 80Gy SIB | 40Fr | Varian | 125 kV | 47 | 2 mm | 512 | 512 | 0.908\0.908 |
|  | 3 | 80Gy SIB | 40Fr | Varian | 125 kV | 37 | 2 mm | 512 | 512 | 0.908\0.908 |
|  | 4 | 80Gy SIB | 40Fr | Varian | 125 kV | 37 | 2 mm | 512 | 512 | 0.908\0.908 |
|  | 5 | 80Gy SIB | 40Fr | Varian | 125 kV | 33 | 2 mm | 512 | 512 | 0.908\0.908 |
|  | 6 | 80Gy SIB | 40Fr | Elekta | 120 kV | 16 | 1 mm | 410 | 410 | 1\1 |
|  | 7 | 80Gy SIB | 40Fr | Elekta | 120 kV | 16 | 1 mm | 410 | 410 | 1\1 |
|  | 8 | 80Gy SIB | 40Fr | Elekta | 120 kV | 16 | 1 mm | 410 | 410 | 1\1 |
|  | 9 | 60Gy | 30Fr | Elekta | 120 kV | 16 | 1 mm | 410 | 410 | 1\1 |
|  | 10 | 50Gy SIB | 25Fr | Elekta | 120 kV | 16 | 1 mm | 410 | 410 | 1\1 |

Table 2: Details of dose prescriptions and imaging device manufacturers for each anatomical site.
